# Phytochemical Analysis and Various Biological Activities of the Aerial Parts of *Scrophularia Atropatana* Growing in Iran

**DOI:** 10.22037/ijpr.2019.1100782

**Published:** 2019

**Authors:** Solmaz Asnaashari, Abbas Delazar, Elham Safarzadeh, Hamed Tabibi, Saeed Mollaei, Ali Rajabi, Parina Asgharian

**Affiliations:** a *Biotechnology Research Center, Tabriz University of Medical Sciences, Tabriz, Iran.*; b *Drug Applied Research Center, Tabriz University of Medical Sciences, Tabriz, Iran.*; c *Department of Pharmacognosy,* *Faculty of Pharmacy, Tabriz University of Medical Sciences, Tabriz, Iran.*; d *Department of Microbiology and Immunology, School of Medicine, Ardabil University of Medical Sciences, Ardabil, Iran.*; e *Student Research Committee, Tabriz University of Medical Sciences, Tabriz, Iran.*; f *Phytochemical Laboratory, Department of Chemistry, Faculty of Sciences, Azarbaijan Shahid Madani University, Tabriz, Iran. *; g *Department of Animal Biology, Faculty of Natural Sciences, University of Tabriz, Tabriz, Iran.*

**Keywords:** Scrophularia atropatana, Phytochemical analysis, Biological activities, GC-MS analysis, MTT assay

## Abstract

*Scrophularia atropatana *(*S. atropatana*)*, *an Iranian plant belonging to the family of Scrophulariaceae, was assigned for its chemical compositions and biological activities of essential oil (EO) and extracts of the aerial parts of the plant during the flowering stage. Combination of GC-MS and GC-FID was assessed for analyzing the chemical compositions of the EO from the aerial parts of *S. atropatana*. Furthermore, Brine shrimp lethality test and DPPH assay were performed to evaluate general toxicity and free-radical-scavenging properties, respectively. Furthermore, anti-proliferative and antimicrobial activities were assessed by MTT assay and disc diffusion methods correspondingly. Additionally, all the potent samples (extracts) and its fractions in the MTT assay were further studied for the presence of various compounds by GC-MS apparatus*.* MeOH extract and 40% sep-pak fraction indicated high amounts of total phenolic (TPC), total flavonoid content (TFC), and antioxidant properties. In the case of general toxicity, among the extracts, dichloromethane (DCM) extract showed noticeable effect. Furthermore, DCM extract was indicated potent ability to eliminate breast tumor cells and minimum efficacy on normal cells. Anti-microbial activity of all samples was ignorable. The potent extracts and fractions which had more anti-proliferative activity were further elucidated by GC-MS and showed high amounts of Alkanes and fatty acids. In the case of EO constituents, non-terpenoids were the major compounds. To sum up, it seems BSLT could be a good preliminary approach for evaluating the cytotoxicity in MCF-7 cell line. Additionally, antioxidant activity, TPC, and TFC contents of all samples were in consistent with each other.

## Introduction

Malignant neoplastic cancer is regarded as one of the most common chronic diseases worldwide and is responsible for several deaths annually (according to a WHO report). Many cancerous patients are resistant to common and conventional strategies such as surgical removal, radiotherapy as well as chemotherapy approaches. Conversely, the serious side effects and patient complications of the aforementioned methods have encouraged scientists to discover new methods and therapeutic drugs for increasing the quality of life, survival rate, and preventing side effects. Since ancient time, natural compounds as complementary sources have been of major interest for various diseases such as cancer (1-3). Hence, many natural products such as vincristine, vinblastine, and others, as potential novel anti-tumor agents, have been isolated from herbal sources or other related sources. Moreover, herbal remedies illustrate acceptable anti-proliferative activities on cancerous cells without causing unfavorable effects on the normal cells. For instance, one of these herbs which should be noted is *Scrophularia atropatana* (*S. atropatana*) L. This plant is an important member of the Scrophulariaceae family, locally known as «*gole meimuni ye Azari» *and occurs mainly in Central Europe, Asia and North America, especially in the Mediterranean area (4, 5). *Scrophularia* genus is represented in the Iranian Flora by 42 species. Previous phytochemical reports on other species of *Scrophularia* have revealed that this genus is known to produce diverse natural compounds including iridoides, phenyl propanoides, flavonoids, saponins, and terpenoids. Other studies on the essential oil compositions of some species of this genus have demonstrated the presence of terpenoid structures, linear hydrocarbons, and their derivatives (4, 6). Hence, this genus because of its already mentioned major constituents, has been holding a place of value in herbology with some known medical properties such as anti-inflammatory, anti-bacterial, cardiovascular, diuretic, protozoacidal, fungicidal, cytotoxic, anti-nociceptive, and wound healing benefits (7-13). In continuation of our scientific works on the analysis of the volatile and non-volatile components of the different plants along with their biological activities (8, 14-19), no report was found on the phytochemicals of *S. atropatana* to date. Thus, we have now evaluated the volatile constituents along with the cytotoxic activity against cancerous and non-cancerous cells, from the aerial parts of the plant that is grown in the Azerbaijan province of Iran. 

## Experimental

*Chemicals*

3(4,5-dimethylthiazol-2-yl) 2,5-diphenyl-tetrazolium bromide (MTT; Sigma-Aldrich, USA); 2,2-diphenyl-1-picrylhydrazyl (DPPH), rutin, gallic acid, Folin-Ciocalteu reagent, Aluminum chloride, penicillin G-streptomycin and Fetal Bovine Serum (FBS) all from Sigma Aldrich (Germany), RPMI 1640 from Gibco, UK, Phosphate Buffer Saline (PBS), Muller Hinton Agar Medium (MERCK) and Trypsin–EDTA (Gibco, Paisley, UK) were purchased. All other reagents and chemicals were of analytical grade. Solvents used for extraction and tests purchased from Caledon and Scharlau. ELISA plate reader (BioTeck, Bad Friedrichshall, Germany) was used for anti-proliferative test. (DPPH) reagent (C_18_H_12_N_5_O_6_ formula, molecular weight 394) (Sigma-Aldrich, Germany), also were used in this paper.

*Collection and identification of plant material*

The chopped aerial parts of *S. atropatana* were gathered in May 2013, at altitudes above 1600 m from Spiran region in East Azerbaijan province. Voucher specimen after identifying (Tbz- fph- 8962) has been retained at the Herbarium of the Department of Pharmacognosy, Faculty of Pharmacy, Tabriz University of Medical Sciences, Iran. 

*Extraction and Fractionation*

Powdered aerial parts of *S. atropatana *were extracted by Soxhlet to obtain n-hexane (n-hex), dichloromethane (DCM) and methanol (MeOH) extracts, successively. For further investigations, the potent extract (inhibited strongly growth of the malignant cells) was subjected to Vacuum Liquid Chromatography (VLC) method for fractionation (20-23). VLC is a method to obtain different fractions of non-polar extracts. In this method, first VLC hopper was connected to Buchner filter, and then a filter paper was put on the filter. In the next step, the silica gel was loaded into the 2/3 of the tightened hopper. Subsequently, the vacuum pomp was used to compact the silica gel. Another filter paper was placed on the silica gel column. After these preparations, 150 mL methanol, 150 mL ethyl acetate, and 150 mL 10% ethyl acetate (10% EtOAc and 90% n-hex) were passed over the silica gel, respectively. Then, the filter paper over the column was removed. Afterwards, DCM extract was solved in adequate 10% EtOAc in n-hex and then the solution was loaded over the column and then step by step several concentrations of EtOAc in n-hex (10%, 20%, 40%, 60%, 80% and 100%) were passed through the column and were collected in separate containers. 

Moreover, the polar extracts which had high free radical scavenging activity and total phenolic contents were fractionated by Solid Phase Extraction (SPE) method. Two grams of MeOH extract was loaded on a Sep-pak (10 g: C18) cartridge (Waters, Ireland) and eluted by increasing step gradients of MeOH-water mixtures. For removing the solvents, Rotary evaporator (Heildolph, Schwabach, Germany) at a maximum temperature of 45 °C and in a low pressure was used. For further investigations, the samples were stored in a freezer at -20 °C.

*Distillation of plant materials*

Shed-dried plant material of the aerial parts of *S. atropatana* was subjected to hydro-distillation for 3 h using a Clevenger-type apparatus. Resulted essential oil (EO) was subsequently dehydrated using anhydrous sodium sulphate and kept at low temperature (4 °C) in dark until analysis.

*Gas Chromatography-Mass Spectrometry (GC-MS) analysis and identification of compounds*

The analysis of the EO and cytotoxic DCM extract and its potent fractions was performed using a Shimadzu GC-MS-QP5050A fitted with a fused DB-1 capillary column (methyl phenyl syloxane, 60 m, 0.25 mm id, film thickness 0.25 µm). Helium was used as a carrier gas at a flow rate of 1.3 mL/min, as well as split ratio 1.29. Temperatures of the injector and detector were set at 250 °C and 260 °C, respectively. The column temperature was kept at 50 °C for 2 min, and then programmed to increase to 230 °C at a rate of 2 °C/min and then kept constant for 8 min. The MS spectral parameters were as follows: ionization energy 70 eV; ion source temperature 200 °C; quadrupole 100 °C; solvent delay 3 min; EV voltage 3000 v. Qualitative identification of constituents was based on direct comparison of the retention indices, Kovats indices and Mass spectral with those for standard compounds and computer matching with the NIST NBS54K Library, as well as by comparison with published papers (4, 6). Flame ionization detector (FID) was used for calculating relative percentage amounts. The FID detector condition was set on the same GC-MS operational conditions. 

*Antimicrobial assay*

*In-vitro* anti-microbial activity of all samples (n-hex, DCM, MeOH and EO) of *S. atropatana* was assessed against following organisms: Two strains of gram negative species, *Pseudomonas aeroghinosa* (ATCC 9027) and *Escherichia coli* (ATCC 8739), as well as the gram positive species namely *Staphylococcus epidermidis* (ATCC 12228) and *Staphylococcus aureus* (ATCC 6538), and a fungi (*Candida albicans*) (ATCC 10231) which were purchased from the institute of Pasture, Type Culture Collection (Iran). Agar disc diffusion method as the qualitative antibacterial assay was used for this aim. Cultured bacteria in to the Muller Hinton Broth was incubated at 37 °C for 24 h. Then, the centrifuged pellets re-suspended in saline solution to provide an optical density equal to 10^6 ^CFU/mL. Test organisms were cultured in a suitable Muller Hinton Agar Medium. Sterile 6.0 mm diameter discs were impregnated with 50 µL of different test substances. Subsequently, the plates for 30 min were kept in a refrigerator to allow the diffusion of extracts, and then they were incubated at 37 °C for 24 h. The quantitative antimicrobial potency of the samples was assessed by measuring the diameter of the inhibition zones in comparison to DMSO and Amikacin as a negative and positive control groups. All experiments were performed in duplicate and mean ± SD value was calculated (24).

*Anti -proliferative activity*

Malignant and nonmalignant cells including SW480 (colon carcinoma) MCF-7 cells (human breast carcinoma cell line) and L-929 (normal cell line) from Pasture Institute, Tehran, Iran were cultured in RPMI 1640 medium with suitable additives containing 10% Fetal Bovine Serum (FBS), 100 mg/mL streptomycin and 100 units/mL penicillin G. They were incubated at 37 °C in a humidified air/carbon dioxide (95:5) atmosphere. MTT colorimetric assay was used for assessing viability of the cells. The cells in the exponential growth stage were sub-cultured at 1 × 10^4^/well on to 96-well plates (Nunc, Denmark). Then, the cell suspensions were treated with different dilutions of all extracts for 24 and 48 h. Four hours before the end of the time, the medium was replaced with a fresh culture containing 20 μL of MTT solution (5 mg/mL in PBS). Then after this period, the supernatants were removed and 100 µL of DMSO solvent was added to dissolve the formazan crystals completely. Finally, the absorbance of the metabolized MTT production (formazan crystals) was read at 570 nm with microplate reader (BioTeck, Bad Friedrichshall, and Germany). (BioTeck, Bad Friedrichshall, and Germany). The IC_50_ values were defined as the concentration of the samples required to induce a 50% reduction in viability of the cells absorbance and evaluated from a dose-response curve plotted in the SigmaPlot 11 software (25, 26). Methotrexate was used as positive control.

**Table 1:** Free radical scavenging test, Total phenolic content, and total flavonoid content of n-hexane, dichloromethane and methanol extracts of *S. atropatana *aerial parts

**Extracts or fractions**	**Total phenol content (mg g** **-1** **)**	**Flavonoid content (mg g** **-1** **)**	**Antioxidant activity (RC** **50** **; mg mL )** -1 **
MeOH*	68.37 ± 0.06	52.04 ± 0.42	0.143 ± 0.09
DCM*	27.29 ± 1.02	-	0.681 ± 0.02
n-hexane	3.21 ± 0.30	-	0.576 ± 0.008
10% SPE fraction	7.44 ± 0.20	5.60 ± 0.31	1.164 ± 0.20
20% SPE fraction	42.54 ± 0.45	25.66 ± 0.33	0.092 ± 0.00
40% SPE fraction	146.65 ± 2.34	120.12 ± 2.25	0.046 ± 0.00
60% SPE fraction	127.52 ± 0.31	100.23 ± 4.43	0.102 ± 0.00
80% SPE fraction	24.76 ± 0.32	14.23 ± 1.12	0.194 ± 0.02
100% SPE fraction	4.76 ± 0.21	3.05 ± 0.01	0.534 ± 0.26

Experiment was performed in triplicate and expressed as mean ± SD.

*MeOH: Methanol; DCM: Dichloromethan.

**The RC value for quercetin as positive control was 0.003 ± 0.00 mg/mL.

**Table 2:** Results of the MTT assay of Vaccum Liquid chromatography fractions of potent Dichloromethane of *S. atropatana *aerial parts against cancer and normal cells

**Samples**	IC50 Values (µg/mL)				
	**MCF-7**		L929		
	**24 h**	**48 h**	**24 h**	**48 h**
10% VLC	123.7 ± 0.98	113.8 ± 43.7	>500	220.00 ± 32.01
20% VLC	103.59 ± 17.83	94.3 ± 13.42	>500	190.23 ± 15.45
40% VLC	247.5 ± 62.08	205.7 ± 39.88	>500	300.00 ± 46.71
60% VLC	148.85 ± 31.60	208.05 ± 38.53	>500	380.23 ± 49.22
80% VLC	98.105 ± 25.59	88.53 ± 12.85	>500	230.45 ± 56.28
100% VLC	62.29 ± 4.29	60.7 ± 10.79	>500	198.20 ± 43.25
Methotrexate	0.16 ± 0.09 (48 h)		0.24 ± 0.01 (48 h)		

VLC: Vaccum Liquid chromatography.

IC50 value of Methotrexate against SW480, was 0.23 ± 0.02 µg/mL during 48 h.

**Table 3:** Chemical constituents of the essential oil from aerial parts of *S. atropatana*

**Area (%)**	**RT (min)**	**KI** ^(a)^	**Molucular formula**	**Compounds**		**Number**	
0.03	10.29	800	C5H4O2	Furfural		1
0.03	16.40	912	C6H12O	2-Pentanone، 3-methyl		2
0.01	18.42	941	C6H14O	Isobutylmethylmethanol		3
0.12	19.02	950	C7H14	Cyclopropane،1،1،2،3-tetramethyl		4
0.02	19.12	952	C7H14	-1Pentene، 2،4-dimethyl		5
0.14	19.23	953	C7H12O	-1Hepten-3-one		6
0.23	19.80	962	C8H16O	-1Octen-3-ol		7
0.02	20.85	977	C9H14O	2-Pentylfuran		8
0.15	20.96	979	C8H16O	Cyclooctyl alcohol		9
0.10	22.83	1006	C8H8O	Benzeneacetaldehyde		10
0.03	24.35	1026	C10H16	beta.-trans-Ocimene		11
0.08	25.17	1037	C10H16	beta.-cis-Ocimene		12
0.09	26.41	1054	C10H20	1-Octene،3،7-dimethyl		13
0.03	28.16	1077	C10H16	α- Terpinolen		14
2.14	28.51	1082	C9H18O	Nonanal		15
18.82	28.87	1087	C10H18O	Linalool		16
0.28	32.35	1133	C10H18O	2-Decenal، (E)		17
0.20	34.04	1156	C10H22O	1-Decanol		18
0.20	34.30	1159	C10H18O	1-Terpinen-4-ol		19
4.14	35.19	1171	C10H18O	alpha.-Terpineol		20
0.54	36.14	1184	C10H20O	Capraldehyde		21
0.13	36.46	1188	C10H16O	Carvomenthenal		22
0.13	36.87	1194	C10H16O	beta-cyclocitrat		23
1.57	38.03	1209	C10H18O	cis-Geraniol		24
5.68	39.91	1235	C10H18O	1،6-octadien-3-ol،3،7-dimethyl		25
0.26	41.65	1259	C9H18O2	3-Ethylheptanoic acid		26
0.33	41.78	1261	C9H18O2	Nonanoic acid		27
0.15	42.21	1267	C11H20	1،3-Nonadiene،5،5-dimethyl		28
0.11	43.18	1280	C9H10O2	Vinylguaicol		29
0.38	43.68	1287	C10H16O	2،4-Decadienal، (E،E)		30
0.26	45.78	1317	C10H16O2	Lilac aldehyde D		31
0.37	47.30	1339	C11H20O	2-Undecen-1-al		32
0.26	48.18	1352	C10H20O2	n-Capric acid		33
0.79	48.77	1360	C13H18O	2-Buten-1-one1-(2،6،6-trimethyl-1،3-cyclohexadien-1-yl)-، (E)		34
0.12	49.31	1368	C13H20	2-Carene،4-.alpha.-isopropenyl		35
0.09	50.59	1387	C13H26O	Pseudoionone hexahydro		36
0.10	50.66	1388	C13H26O	Tridecanal		37
0.21	51.18	1395	C13H20O	6،8-Nonadien-2-one،6-methyl-5- (1-methylethylidene)		38
0.42	53.35	1428	C13H22O	Dihydropseudoionone		39
0.36	55.55	1462	C13H20O	beta-lonone		40
0.17	57.27	1488	C14H26	cis،cis-5،9-Tetradecadiene		41
0.20	57.40	1490	C13H26O	Tridecanal		42
0.67	60.99	1547	C15H26O	trans-Nerolidol		43
0.14	63.78	1592	C16H32O	Hexadecanal		44
0.18	67.70	1658	C16H34O	1-Decanol، 2-hexyl		45
0.05	68.11	1665	C15H26O	alpha.-Bisabolol		46
0.14	69.84	1694	C15H30O	Pentadecanal		47
0.29	70.18	1700	C17H36	Heptadecane		48
0.48	72.69	1745	C15H30O2	Pentadecanoic acid		49
0.14	75.58	1796	C16H32O	Palmitaldehyde		50
0.13	75.78	1800	C18H38	Octadecane		51
0.23	77.11	1825	C16H22O4	Phthalic acid، diisobutyl ester		52
3.65	77.40	1830	C18H36O	2-Pentadecanone trimethyl		53
0.10	79.60	1872	C18H38O	Hexadecane،1-methoxy-13-methyl		54
0.10	81.55	1909	C17H34O2	Hexadecanoicacid،methyl ester		55
0.66	83.42	1945	C18H36O2	Octadecanoic acid		56
0.20	86.23	2001	C19H40O	Nonadecanol		57
0.07	95.69	2100	C21H44	Heneicosane		58
0.10	89.84	2124	C19H32O2	Linolenic acid، methyl ester		59
2.92	91.06	2125	C20H40O	Phytol		60
2.58	100.52	2223	C22H46	Pentadecane، 8-heptyl		61
0.12	104.42	2400	C24H50	Tetracosane		62
1.09	108.53	2800	C28H58	Octacosan		63
37.15	110.76	3100	C31H64	Hentriacontane		64
0.09	112.47	3600	C36H74	Hexatriacontane		65
	90.77%				Total identified		
	37.11				Terpenoids		
	60.66				Non-terpenoid		

^a ^Compounds listed in order of elution from a DB-1 column.

**Table 4:** Compositions of Dichloromethane extract and its 80%, 100% potent cytotoxic Vacuum liquid chromatography fractions of *S. atropatana *aerial parts

**Samples**	**Compounds**	**RT** **(a)**	**Area (%)**
	Neophytadiene	27.83	4.59
	3,7,11,15-Tetramethyl-2-hexadecen-1-ol	28.74	1.3
	n-Hexadecanoic acid	29.98	4.6
	Menthol	33.27	2.04
DCM extract	9-Octadecenoic acid	33.52	2.47
	n-Nonadecane	40.32	1.47
	n-Octadecane	43.49	3.38
	n-Tetratriacontane	46.82	53.77
	Octacosane	48.69	4.26
	Loliolide	22.13	38.83
80% VLC fraction	Neophytadiene	24.76	32.62
	Octadecanoic acid	26.89	28.55
	n-Pentadecane	11.73	7.24
	Eicosane	16.98	9.58
	Loliolide	22.14	35.27
	n-Heneicosane	23.10	5.71
100% VLC fraction	Neophytadiene	24.77	9.93
	3,7,11,15-Tetramethyl-2-hexadecen-1-ol	25.68	3.47
	n-Hexadecanoic acid	26.94	23.99
	Phytol	30.23	1.98
	1,2 Benzenedicarboxylic acid, ditridecyl ester	37.52	2.83

aCompounds listed in order of elution from a DB-1 column.

DCM: Dichloromethan; VLC: Vaccum Liquid chromatography; VLC: Vacuum liquid chromatography.

*Assay for Total Phenolics Content (TPC)*

Total phenol content was evaluated by modified Folin-ciocalteu reagent method and gallic acid as standard. Concisely, 1 mL of each total extracts (5 mg in 60% aqueous acetone) were mixed thoroughly and shacked with 2 mL of 10% Folin Ciocalteu reagent and 1 mL of 5% aqueous Na_2_CO_3_ (which was prepared by dissolving Na_2_CO_3 _in water) in a volumetric flask. Subsequently, the mixtures were centrifuged in 1200 rpm for 5 min and allowed to incubate for 30 min at 25 ^o^C. Afterwards, absorbance of upper mixture was assessed at constant wavelength 750 nm. The same procedure was applied to different concentrations of Gallic acid solutions as a standard and calibration curve was drawn. TPC was reported as Mg gallic acid equivalent per gram of dried extract (27).

*Estimating of total flavonoid contents by colorimetric assay (TFC)*

Total flavonoid contents of the samples were determined by leading a modified aluminium chloride colorimetric method and also *Rutin* as a standard. Briefly, 2 mL of different extracts and fractions (which were prepared in 80% methanol) were mixed with 400 μL of water, 400 mg Sodium acetate, 183 mg Aluminium chloride and were kept in dark place at 25^ o^C for 30 min for complete reaction. In addition, the absorbance of all solutions was read at 430 nm. Rutin standard (which was prepared in 5-25 µg/mL dilutions) was applied for calibration curve quantitatively. TFC was calculated as Rutin equivalents per gram of the dried plant material (28).

*Brine Shrimp Lethality Test (BSLT)*

BSLT as a simple, low cost, high sensitive, and convenient pharmacologic guide was applied in a laboratory bioassay for screening general toxicity through the assessment of the 50% fatal concentration following the modified Meyer method and previous works (7). Concisely, the hatching eggs which were obtained from Fisher Center, Tabriz, Iran, were prepared in 35% salt water and incubated under well aerated, flask for 48 h. Subsequently, the different herbal extracts were dissolved in DMSO and normal saline for obtaining variable concentration of the samples. (It is worth to mention that ultimate DMSO concentration of did not overpass 0.05%). One milliliter of main prepared sample solution along with 10 mL of sea water was added to each sterile vial. In addition, approximately, 10 nauplii as amateur shrimps were transferred in to the vials and incubated for 24 h. Finally, number of dead nauplii at each dosage were counted as percent of mortality of the extracts. The LC_50 _was estimated using linear regression analysis by Microsoft Excel software.

*Free radical scavenging activity test (FRST)*

Free radical scavenging potential of all test samples was estimated using 2, 2-diphenyl-1-picrylhydrazyl (DPPH) reagent (C_18_H_12_N_5_O_6_ formula, molecular weight 394) (Sigma-Aldrich, Germany) which was adopted with slight modification (29, 30). DPPH solutions (0.08 mg/mL) for non-polar and polar extracts were prepared in chloroform and MeOH, respectively. Different concentrations of extracts and fractions (5 × 10^-1^, 2.5 × 10^-1^, 1.25 × 10^-1^, 6.25 × 10^-2^, 3.13 × 10^-2^ and 1.56 × 10^-2 ^mg/mL) were obtained by serial dilutions, then mixed with 0.08 mg/mL DPPH solution. The mixtures were incubated for 30 min in 25 °C for complete reaction. The UV absorbance of all samples was measured at a constant 517 nm. The reduction of free radical capacity was calculated by the following formula:

R% = (A _Blank_ – A _Sapmle_)/A _Blank_) × 100                   (1)

Subsequently, 50% inhibition capacity value was resulted from the graph plotting reduction percentage against different concentrations of the extracts. Quercetin was used as a positive control. All of the experiments were followed in the same manner for positive control. The experiment was repeated in triplicate.

*Statistical Analysis*

All experiments were conducted in triplicate measurements and presented as the mean ± SD. Data were analyzed by Microsoft Excel and SigmaPlot 2010. The IC_50_ value was calculated from nonlinear regression analysis.

## Results

In the current research, free radical scavenging activity, TPC, TFC, anti-proliferative activities and GC-MS analysis of three different extracts and also EO obtained from the aerial parts of *S. atropatana* were determined and the results were illustrated in Tables 1-4.

Antioxidant characteristic of the* S. atropatana *EO, extracts and its fractions:

The findings of antioxidant activity of the EO, extracts, and Sep-pak fractions of *S. atropatana *are shown in Table 1. A low cost and preliminary method to assess the free radical scavenging potency of the samples is based on a colorimetric assay. In the current assay, MeOH extract and its fractions indicated free radical scavenging activities in a concentration-dependent manner. RC_50 _values of MeOH extract, 40% and 20% Sep-pak fractions (0.143 ± 0.09, 0.04 ± 0.00 and 0.092 ± 0.00 mg/L, respectively) in comparison to the quercetine as a reference control (RC_50 _= 0.003 ± 0.00 mg/mL) indicated a moderate activities.

*Total phenols content (TPC)*

Total phenolic constituents were calculated quantitatively as standard gallic acid equivalents, using Folin- Ciocalteau`s method. Our findings in Table 1 illustrated that MeOH extract, 40% and 60% MeOH-Water Sep-pak fractions with (68.37 ± 0.06, 146.65 ± 2.34 and 127.52 ±0.31 mg GAE/g of samples, respectively) were superior to the phenolic content of the other extracts and fractions.

*Total Flavonoids Content (TFC)*

The TFC of the samples were evaluated using aluminum chloride reagent assay (Table 1). The amount of flavonoids in all samples were expressed as rutoside equivalent (as the standard flavonoid) in mg g^-1 ^dry samples. It is notable that the TFC content of MeOH extract, 40% and 60% Sep-pak fractions (52.04 ± 0.42, 120.12 ± 2.25 and 100.23 ± 4.43 mg rutin equivalent in 1 g of dried powdered plant material, respectively) encompass the significant amount of flavonoids than the other samples. 

*General toxicity*

Preliminary Brine Shrimp lethality bioassay was used for comparing the cytotoxic activity of the extracts with positive control (Podophyllotoxin LC_50 _= 2.69 µg/mL). Results illustrated that the DCM and MeOH extracts with LC_50 _values of 0.064 and 0.271 µg/mL respectively, showed moderate effects. In terms of n-hex extract and EO no significant effects were observed.

*Cytotoxic activity*

Based on the BSLT findings, the DCM and MeOH extracts of *S. atropatana *illustrated considerable effects in comparison to n-hex sample. So, potent samples were selected for further anti-proliferative investigations on both SW-480 and MCF-7 cell lines as the cancerous cells and L-929 as the normal cell line during 24 and 48 h period (Table 2). 

Hence, MTT method was accomplished for assessing the cytotoxic activity. IC_50_ values for more elucidating the consequence were indicated in Table 2. Based on our previous published data against MCF-7 cell line (31), IC_50_ value for DCM extract against MCF-7 cell line was 223.00 and 114.70 µg/mL during 24 and 48 h. However, MeOH extract illustrated minimum growth inhibition activity on SW480 during 24 and 48 h (674.10 ± 45.30 and 557.20 ± 15.02 µg/mL) compared with DCM extract in 24 and 48 h (420.25 ± 25.15 and 390.31 ± 22.35 µg/mL), respectively. According to the results, DCM extract clarified more cytotoxic effect on both SW-480 and MCF-7 cell lines in comparison to MeOH extract at 24 and 48 h incubation. Moreover, the amount of IC_50 _value of DCM extract on MCF-7 cells at 24 h and 48 h was lower than SW-480 cell line. On the other hand, DCM extract revealed more remarkable cytotoxicity in a time and dose dependent manner. Beside, MCF-7 cell line was selected for further evaluation by VLC fractions of DCM extract. Among the fractions, 80% and 100% VLC fractions (with IC_50 _= 88.53 ± 12.85, 60.7 ± 10.79 (µg/mL), respectively), revealed considerable effects on breast cancer cell line at 48 h. Interestingly, L-929 as a normal cell line was not considerably impressed by MeOH and DCM extracts) after 24 (IC_50_ values > 500) and 48 h (321.49 ± 32.24 and 264.30 ± 11.32 µg/mL), respectively. This means that DCM extract and its 80%, 100% VLC fractions inhibit growth of cancer cells with no harmful effect on normal cells. No anti-proliferative activity has been observed via essential oil.

*Antimicrobial activity*

No antimicrobial activity of the test specimens was observed against studied microorganisms.

*GC-MS results*

GC-MS analysis results of essential oil and potent DCM extract, 80% and 100% VLC fractions of the aerial parts from *S. atropatana *have been indicated in Tables 3 and 4. Hentriacontane (37.15%) and Linalool (18.82) (as Main components of EO), n-Tetratetracontane (53.77%) (Main compound in DCM), Loliolide (38.83 and 35.27%) (As a major ingredient in 80% and 100% VLC fractions, respectively) were in high amounts in the mentioned samples.

## Discussion

Colon cancer as a malignancy of the digestive system and breast cancer are the most common and prevalent types of malignancy in men and women, respectively. According to the statistics of world cancer, colon cancer is the third while breast cancer is the second most common cancers worldwide and in some cases it is associated with major mortality and morbidity. The incidence of both types of cancers is alarmingly rising both in developing and developed countries. Environmental factors play a critical role in boosting the genre of cancers (1, 2). Nowadays, because of the inefficiency of conventional therapeutic methods, numerous efforts have concentrated on finding safe constituents with high efficiency and minimum unwanted side effects, to ameliorate the various types of cancer. In this regard, the use of natural origin products with low unfavorable effect has made them high interest and the most popular tool (1, 2 and 31) in recent years. 

In the current study, BSLT and FRST as valid, low cost, easily mastered laboratory bioassays, were applied to assess the toxicity and free radical effects of different extracts of *S. atropatana* specimen, respectively (32, 33). Although, among the extracts, the DCM and MeOH extracts of *S. atropatana* had considerable effects against *A. salina.* The non-polar extract (n-hex) and EO of plant has the lowest capability to inhibit *A. salina* proliferation. Subsequently, two malignant cell lines (MCF-7, SW480) and a normal cell line (L929) were applied for further evaluation of the *in-vitro* cytotoxic potential of potent DCM and MeOH extracts, using the MTT assay. The cytotoxic activity of samples was based on the reduction of tetrazolium MTT (yellow) to formazane dye (purple). Hence, the amount of formazan showed the number of viable cells. In our previous study, we evaluated the cytotoxic effects of different extracts of *S. atropatana* on the MCF-7 cell line. The MTT results showed that during 24 h, different concentrations of samples did not exert any noticeable anti-cancer effect (34). Correspondingly, after 48 h, DCM and MeOH specimens significantly increased the anti-proliferative effects on MCF-7 and showed no significant impact on the L929 normal cells, in a dose and time-dependent manner. The IC_50_ amount of DCM is lower than the MeOH extract. In a recent study, the anti-proliferative activity of all extracts was assessed against the SW480 cell line as well. The IC_50_ amounts on the SW480 in comparison to the MCF-7 cell line were high. In other words, the ability of the extracts to eliminate the colon tumor cells in comparison to breast cancer was negligible (it is worth mentioning that the DCM extract not only in the MCF-7 cell line, diminished the cancer cells but also, in the SW480 cell line it eliminated the colon cancers). It appears that the MTT results are supported by the BSLT findings. Conversely, in our present study, there is a parallel relationship between the cytotoxicity of BSLT and the MTT assay which is consistent with the reports which illustrated the direct correlation between these techniques (7). Therefore, it appears that BSLT could be an appropriate preliminary method for predicting cytotoxicity in the MCF-7 cell line in this plant. In this regard, based on the remarkable cytotoxic property of the DCM extract on the breast cancer cell line, six fractions of DCM extracts were prepared for further elucidations. It was found that, 80% and 100% fractions at 24 h and 48 h showed significant anti-tumor effects on breast cancer cells with no noticeable inhibitory impact on L929 cells. 

The results revealed the accumulation of cytotoxic components in polar VLC fractions. Extra researches have illustrated the anti-proliferative activity of various species of the *Scrophularia* genus (7, 34-36). The findings of the present study are in line with the studies of Giessrigl B *et al.* (35), Asgharian P *et al.* (7), and Valiyari S (36). In all studies, DCM and MeOH extracts significantly suppressed the growth and replication of different cancer cells, especially the MCF-7 cells by inducing apoptosis. The preliminary phytochemical analysis of the DCM extract in our previous study, illustrated the presence of cardiac glycosides, sterols and terpenoids (7, 19). Moreover, the cytotoxic activity of the mentioned secondary metabolites has been reported by previous published papers (16, 37-39). To predict the volatile chemical constituents of the potent DCM extract and its fractions, GC-MS analysis was applied. In all samples, hydrocarbons such as n-Tetratriacontane and Loliolide were the main components which showed growth inhibition on cancerous cells in previous literatures (40-42). Moreover, in the recent study, the relationship between TPC, TFC as well as the free radical scavenging ability of different extracts of *S. atropatana* were evaluated. Among the extracts, the MeOH extract, 40% and 60% Sep-pak fractions demonstrated potent free radical inhibition with high amounts of phenolic contents. Although, the free radical scavenging activity of the MeOH extract was high; the anti-neoplastic activity of this extract in comparison to the DCM extract was negligible. While, the general toxicity and cytotoxic activity of the extracts were in parallel, TPC, TFC, and free radical scavenging activities of the extracts were also in line with each other. 

It appears that the mechanism of cytotoxic activity of *S. atropatana* was different from the radical scavenging activity and total phenolic contents. In the case of the chemical constituents of EO, non-terpenoids (Hentriacontane) and terpenoids (Linalool) were in high amounts, respectively. The results were consistent with our previous published work which indicated that linalool was the main common compound in *S. frigida* and *S. subaphylla* (4, 6). The agar disk diffusion method was used to screen the anti-microbial activity of the all samples. None of the extracts and EO illustrated growth inhibition on microorganisms. The next step of the research should be concentrated on the isolation of pure anti-neoplastic compounds of potent fractions and evaluation of its anti-proliferative mechanisms.

## Conclusion

Generally, the current survey proposed that the presence of some bioactive components in DCM and MeOH samples inhibit the growth of cancerous cells and also demonstrates the free radical scavenging activity with high contents of TPC and TFC in MeOH, respectively. It seems that the mechanism of cytotoxic activity of *S. atropatana* was different from the radical scavenging activity and total phenolic contents. 
